# Irreplaceable Role of Amendment-Based Strategies to Enhance Soil Health and Disease Suppression in Potato Production

**DOI:** 10.3390/microorganisms9081660

**Published:** 2021-08-03

**Authors:** Jianjun Hao, Katherine Ashley

**Affiliations:** School of Food and Agriculture, University of Maine, Orono, ME 04469, USA; katherine.ashley@maine.edu

**Keywords:** soilborne disease, microbiome, biological control, organic matter

## Abstract

Soilborne diseases are a major constraining factor to soil health and plant health in potato production. In the toolbox of crop management, soil amendments have shown benefits to control these diseases and improve soil quality. Most amendments provide nutrients to plants and suppress multiple soilborne pathogens. Soil amendments are naturally derived materials and products and can be classified into fresh or living plants, organic or inorganic matters, and microbial supplements. Fresh plants have unique functions and continuously exude chemicals to interact with soil microbes. Organic and inorganic matter contain high levels of nutrients, including nitrogen and carbon that plants and soil microorganisms need. Soil microorganisms, whether being artificially added or indigenously existing, are a key factor in plant health. Microbial communities can be considered as a biological reactor in an ecosystem, which suppress soilborne pathogens in various mechanisms and turn soil organic matter into absorbable forms for plants, regardless of amendment types. Therefore, soil amendments serve as an energy input, nutrient source, and a driving force of microbial activities. Advanced technologies, such as microbiome analyses, make it possible to analyze soil microbial communities and soil health. As research advances on mechanisms and functions, amendment-based strategies will play an important role in enhancing soil health and disease suppression for better potato production.

## 1. Introduction

The potato (*Solanum tuberosum* subsp. *tuberosum* and *S. tuberosum* subsp. *andigena*) is the third most important food crop for human consumption and provides healthy nutrients to the global population [1,2]. In order to enhance potato health, providing required nutrients to the plant and reducing soilborne diseases are the most important aspects to consider. The consumable part of the potato, the tuber, is also commonly used as a “seed” for vegetative propagation, which requires a high volume of nutrients. Compared to many other crops, potato production requires intensive tillage, resulting in minimal plant residues left over and the rapid loss of nutrients in fields. Therefore, it is important to input organic materials or fertilizers into soil to maintain fertility after each potato crop [1]. On the other hand, since potato tubers are exposed to the soil for almost the entire period of growth, from mother tubers to newly produced daughter tubers, the potato is challenged by many soilborne pathogens [1,2]. The resulting diseases directly impact the quality and yield of potatoes.

There are at least 85 known diseases that can cause the loss of potato production to different extents, and half of them are soilborne [3,4]. The number and type of pathogen is not the only aspect which complicates potato production, as many potato diseases are complex and multifaceted. Examples of some diseases are oomycete diseases such as pink rot [5], late blight [6] and Pythium leak [7], fungal diseases such as Rhizoctonia stem rot [7], and bacterial diseases such as common scab [8,9]. Various organisms can cause the same or similar disease symptoms. For example, dry rot can be caused by over 10 different species of *Fusarium* [10], blackleg and soft rot is caused by dozens of bacteria under the *Dickeya* and *Pectobacterium* genera [11], and diversified *Streptomyces* spp. are frequently identified causing potato common scab [12]. Additionally, some pathogenic microorganisms can interact, suppress, or promote others in multiple ways. For example, early death in potatoes is caused by *Verticillium dahliae,* but lesion nematodes exacerbate the disease symptoms [13]; *Spongospora subterranea* f. sp. *subterranea* is the causal agent of powdery scab [14], but is also a vector of mop top virus [14]; and *Dickeya dianthicola* and *Pectobacterium parmentieri* can synergistically enhance the overall severity of blackleg and soft rot in the field [15].

Most soilborne pathogens can survive in soil for a long time because they have specialized survival structures, such as sclerotia, oospores, and chlamydospores [6,7,16,17]. These structures help them to survive through winter months and periods without available hosts. As a result, leaving the field fallow may not quickly reduce the population of pathogens, and more aggressive actions may need to be taken to control soilborne diseases. In disease management, many strategies have been applied and shown their effectiveness in modern production, such as the development of resistant varieties, soil fumigation, and chemigation [3,4,18,19]. However, limited resistant germplasm of potato cannot satisfy the need for diversified varieties for controlling all facultative and soilborne pathogens [20,21,22], and pesticides can be a good option, although not for all fungal pathogens [23]. Pesticides also bring ecological, sustainability, social impacts, and public concerns [24,25]. To keep potato production sustainable, cultural practices, such as soil amendments have been extensively studied and applied [19,26,27,28,29,30,31,32]. In this review, we will discuss amendment-based strategies, with emphasis on plant- and microbe-related products in the control of soilborne diseases. 

## 2. Soil Health and Plant Health—A Holistic Approach

**Figure 1:**
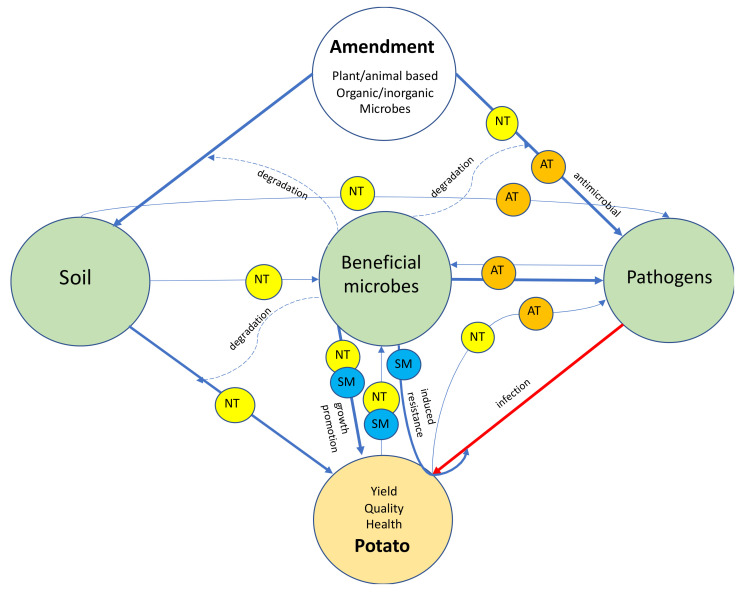
Schematic diagram showing the relationships among potatoes, soil, soil microbiome (beneficial microbes and pathogens) and soil amendments, placed in larger circles. Various compounds (small circles) are produced from corresponding materials, represented by antimicrobial substances (AT); small molecules (SM such as enzymes, proteins, signal molecules, toxins, hormones, volatile organic compounds, etc.), and nutrients (NT). Solid lines in blue with arrows indicate activities by providing key materials, and level of activity is measured by the width of the line. The red solid line indicates plant infection caused by soilborne pathogens. Dashed lines indicate a biological process of materials in the system.

Soil health in agricultural settings can be defined as the continued capacity of soil to function as a vital living ecosystem that sustains plants, animals, and humans, suggested by the US Department of Agriculture (https://www.nrcs.usda.gov/wps/portal/%20nrcs/main/soils/health/, accessed on 15 July 2021) [33]. Soil is a dynamic and living ecosystem. The integrity of soil is highly affected by soil microorganisms, which regulate soil quality and fertility, and modify soil health [29,33]. Soil is a medium for holding plants, a reservoir of nutrients, a habitat for a large diversity of microorganisms, and a vessel for microorganisms to process organic matter into usable compounds for plants [19,34]. Soil microbial communities include many plant pathogens and also beneficial microorganisms that suppress plant pathogens directly through antagonistic activities, or by inducing plant resistance [4,35,36]. Furthermore, numerous signal molecules from plants and soil microbes mediate the relationships between microbe–microbe and microbe–plant networks [19,25]. Put simply, soil is where plant pathogens survive and where disease control can also be found (Figure 1).

Managing soilborne pathogens starts from improving soil health [19,25,29,37,38,39,40]. Soil health forms the foundation and conditions for plant health, whereas plant health is the goal of crop production and an indicator of soil health. In potato production, high yield and high quality of harvested tubers can be a measurement of plant health [1,39]. The definition of plant health varies depending on different interests and disciplines [41], and has been extended to the association with human health and ecosystem services beyond sustainable plant production [41]. In plant disease-focused subjects, plant health can be considered as the status of a plant reaching its full genetic potential, free of biotic constraints (such as insects and diseases) and in optimal biotic conditions (such as environments and nutrients). In this context, there are two major factors that impact plant health. The first factor is soil physical and biochemical properties, such as pH, organic matter, carbon and nitrogen. Optimal conditions enhance plant potential growth, but lack of some of these elements can cause stress to plants and therefore reduce their growth and health. The second factor is biological and microbial components, particularly soilborne pathogens. High populations of pathogens are associated with disease or reduced production, but low populations or no pathogens can assure a good condition for plant growth and productivity.

For long-term considerations in managing soilborne diseases of potato, a holistic system has been practiced [42]. This requires all soilborne diseases to be managed, soil health which is sustainable, plants to have high yield and high quality, and all of these to be leading toward food and environmental safety. This approach can be expressed as a “plus and minus” system. For gaining a maximal potential of plant growth, one inputs required physical and biological elements to soil to reach optimal conditions (“plus”); and reduces, suppresses, and eliminates soilborne pathogens (“minus”). Therefore, one measurement of soil health is the enhancement of plant productivity and quality food production, while the other is the reduction, elimination or suppression of soilborne pathogens.

As such, applying soil amendments is a top priority [26,27,31,43]. Soil amendments can be an aspect of holistic disease management, integrated pest management (IPM), and biological control [44]. There are many research programs and projects that are developed based on this concept. For example, the Potato Sustainability Initiative involves a broad collaboration of growers, industries, consumers and the National Potato Council, which aims to improve soil health and the sustainability of potato production [19]. In the United States, a multi-state research project was funded by USDA-NIFA to find non-chemical long-term strategies for controlling potato diseases by using cover crops and soil amendments (https://potatosoilhealth.cfans.umn.edu/, accessed on 15 July 2021).

## 3. Soil Amendment for Disease Management—From Practice to Promise

Soil amendments are various materials added to soil in order to modify physical, biochemical, and microbial properties. If the soil microbiome functions as an engine for biological activities, then soil amendments are the fuel (Figure 1). Amendments can be categorized as plant-based amendments, organic and inorganic matter amendments, and microbial amendments. Plant based amendments are living plants such as rotation crops, or partially living plant materials which are later integrated into the soil or killed, such as green manures and cover crops [43,45,46]. Microbial amendments are artificially characterized microorganisms that are applied in large volume to soil.

Historically, organic amendments, such as composts, have long been used in agriculture for fertility purposes, without knowing much about their mechanisms [47]. The functions of amendments vary and are highly dependent on what is being used [48]. Collectively, soil amendments can improve soil health by reducing moisture loss through evaporation and runoff, inhibiting weed growth, increasing soil organic matter, suppressing soilborne diseases, promoting plant growth, and enhancing plant resistance to diseases [39,49].

### 3.1. Plant-Based Soil Amendments—A Microbial Recruiter 

**Figure 2:**
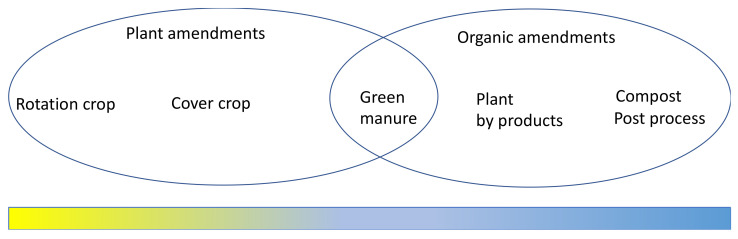
Schematic diagram of plant-based materials used as soil amendments and their stages in plant cycle. The bar on the bottom shows plant stages, changing from living plants (yellow) changing to decomposed compounds (blue).

Living plants used during non-potato growing periods can be considered a type of soil amendment if they are a non-host of major diseases [43,45,46]. Since the products used are living plants, or at least part of their life is in fresh or living form, they provide rich organic matter to soil through root exudates and residual biomass of plant tissues, which are biologically active. Typically, plants are used as rotation crops, cover crops, or green manures, and the key factor is that the materials are used fresh (Figure 2). After harvest, any of the plant-derived products and residues will be considered as an organic amendment. Plant-based amendments noticeably increase potato yield due to a high volume of biomass input and in the meantime diseases can be suppressed at various levels [50,51].

A rotation crop is usually a cash crop, and it takes one complete season to finish the growth cycle. They can be arranged to be grown for one season in a two-year rotation, or over multiple years [47,51]. Usually, longer duration of rotation cycles results in better disease control [35,47,51,52]. Crop rotation has multiple benefits, as it impacts physical soil properties and reduces soil erosion. Equally importantly, it reduces soilborne pathogens by disease-suppressive or non-host crops, and enhances beneficial microorganisms and overall soil health [39,51,53,54]. Commonly used crops for rotation in potato production include, but are not limited to, Brassica crops (e.g., horseradish, mustard, broccoli, turnip, canola, radish, and wasabi), grain crops (e.g., barley, wheat, oat, maize, Sudan grass, ryegrass and rye), legumes (alfalfa, clover, peas, vetch), and beets (Table 1, Figure 2). Mungbean and sunn hemp used as a non-host rotation crop reduce *Streptomyces scabies* population in soil and enhance rhizospheric soil microflora, especially the antagonists fluorescent pseudomonads and *Trichoderma* spp. through root exudates [55], and sunn hemp has an allelopathic effect in suppressing soilborne diseases and nematodes [38]. Broad beans affect soil microbial communities, diversity and crop yield in a long-term continuous potato cropping field [56]. In summary, rotation crops are selected based on the criteria that they are (1) non-host cash crops; (2) economically viable options; (3) crops containing substances such as antimicrobial activities [22,27,53,57].

Cover crops, such as barley and ryegrass used in potato to suppress *Rhizoctonia solani*, are normally planted before or after potato planting and are not harvested for yields (Figure 2). In this way, the soil is covered almost year-round to prevent erosion while also adding organic matter to soil. Rotation and cover crops share some common characteristics: both are living plants which produce some active compounds that affect soil and the soil microbiome [19,46,53,70,71].

Green manures fall between plant-based amendments and organic amendments (Figure 2). They are grown in the first part of the growing season but chopped and freshly incorporated into soil before maturity; therefore, both rhizospheric activities and organic materials provide function [39,49]. Green manure has been used for at least 2000 years [72]. Since green manures have a period growing in the field, they share some features with rotation crops through root exudates. However, the main function of green manures is the use of the canopy biomass [43,66]. Large volumes of biomass of green manures contain high contents of organic matter, and organic nitrogen is associated with disease suppression and yield increase in potato [16,73,74]. Carbon sources provide energy for soil microorganisms that indirectly affect the soil condition or soil health for disease suppression as a consequence of microbial community changes [44]. After being incorporated in soil, the top part of plants become residual products. Since plants used as a green manure have two phases, including root growing systems and freshly obtained organic matter, they share common characteristics of both types of materials. Collectively, plant-based amendments have various functions, such as serving as a non-host, having allelopathic effects, producing specific toxic compounds to inhibit pathogens, and recruiting beneficial microorganisms [65,75,76].

Mechanisms of plant-based amendments include direct and indirect activities. Non-host and allelopathic effects of plants are sources of disease suppression. Soilborne pathogens such as *Colletotrichum coccodes,* the causal agent of black dot, that depend highly on the host, can be eliminated by extended periods without the potato host [51]. However, most pathogens can sustain at a certain level without the presence of hosts [77], so more aggressive actions should be taken. The application of plant-based amendments can suppress soilborne pathogens by interrupting their life cycles and reducing their survival and accumulation [78]. For example, soybean used as green manure significantly reduced common scab build up [66], and potato rotated with red clover or barley undersown with red clover reduced pink rot [63] and *Rhizoctonia*-caused diseases [54]. In non-potato crops, a longer period (years) of rotation helps to reduce pathogen populations in soil. For example, a three-year rotation is better than two-year rotation for disease control [54,66]. Although black dot can be reduced by crop rotation, it does require at least five years out of potato [51], which may not be practical.

Plants produce antimicrobial substances through root exudates, which are the most important factor in cover and rotation crops in disease suppression [79]. The exudates contain various chemicals that can attract and affect soilborne pathogens and beneficial microorganisms. Susceptible varieties of potato may exude some chemicals that attract and encourage the growth of soilborne pathogens for infection [80]. In susceptible potato varieties, these molecules affect all oomycetes and some fungi. This can significantly change the soil microbiome. In contrast, resistant varieties of potato and cover or rotation crops release chemicals in opposite ways and progressively change the soil biochemistry and microbiome. Plant exudates promote special groups of bacteria that suppress *Streptomyces scabies* and other pathogenic bacteria that cause common scab of potato [73]. Disease suppression has been frequently observed on common scab if potatoes are continuously planted for years [81]. It is not surprising that continuous cropping of potato can increase the incidence of common scab each year for several years because potato is susceptible to the pathogen *S. scabies*. However, the same biological environment can increase the population of other microorganisms, including antagonistic bacteria against *S. scabies,* particularly non-pathogenic *Streptomyces* spp. that play an important role in inhibiting *S. scabies*. Both pathogenic and non-pathogenic *Streptomyces* spp. may require very similar nutrients for living, and their ratio of populations may change after years of competition regulated by plants; therefore, the disease may progressively decline [66,81]. The driving forces of this microbial dynamic are the root exudates from the rhizosphere [79,82,83,84].

Similarly, plant amendments, in the form of rotation crops, interact with soil by releasing chemical compounds from root exudates and physical root systems. Microbial taxa in the rhizosphere are highly dependent on the plant taxa [85]. Host plants harbor different endophytic and rhizospheric microbiomes, which in turn contributes to plant resistance [86]. It has been extensively proven that the composition of the rhizosphere microbiome can be influenced not only by species, but even plant genotypes [85,86,87]. Therefore, selecting a specific crop for rotation can target certain groups of soilborne pathogens to be suppressed and beneficial microorganisms to be enhanced. Plant diversity supports microbial biodiversity through root exudates and rhizo-deposition [42,83].

### 3.2. Organic and Inorganic Amendments—The Fuel of The “Microbial Engine”

Although inorganic amendments are important for disease suppression and fertilization [88], we will focus more on organic amendments in this review. Organic amendments have been used for more than 2000 years [89]. They are classified as: (1) by-products of animals (biosolids, meat meal, bone meal, animal manure, biosolids, feather meal, poultry and swine manure, etc.) [32,61]; (2) by-products of plants (soy meal, sphagnum peat, wood chips, grass chippings, straw, sawdust and wood ash, etc.); and (3) produced and processed materials (biochar, compost, etc.). After being applied to soil, most organic amendments need to be further decomposed and turned into various small molecules through a process of microbial degradation before taking effect, although some of them are active elements that affect plant growth and soilborne pathogens immediately. Compost is unique because it is ready to use, as most organic matter that it is composed of is already degraded [90].

Functions of organic amendments include, but are not limited to physical disturbance and interruption of pathogens, disease suppression, providing nutrients to plants, and improvement of soil properties. Most organic amendments contain a high level of nitrogen and carbon contents [31]. However, the volume of application of organic amendments is crucial as large amounts of amendments added to soil may change soil properties, such as pH and soil structure.

Different organic amendments contain different bioactive chemicals. These can be readily available or derived from chemical degradation, which most likely involves transformative action by soil microorganisms. Although these amendments have been used extensively, knowledge of their mechanisms is relatively limited. Lazarovits and his group have found that organic amendments containing high nitrogen normally release toxic compounds such as ammonia and nitrous acid through a series of biochemical and microbial activities [31,32]. Through biological activity, ammonium is converted to ammonia, which is a volatile gas that functions in a similar way to a fumigant and inhibits pathogens [49,91]. Various types of organic materials belonging to this group can suppress soilborne pathogens with high efficiency due to these toxic compounds. For example, animal by-products such as blood meal, bone meal and fish meal, used as soil amendments can reduce viable populations of *V. dahliae*, which in some cases can be reduced to a non-detectable level [92].

In the study of animal manure, volatile fatty acids (VFA), such as acetic acid, have been found to be responsible for the suppression of certain diseases [61,93]. Liquid swine manure can kill *V. dahliae* populations in soil in one to two days after application [49]. Young composts contain high concentrations of similar acids found in animal manure [49]. Fish emulsion can suppress *Verticillium* wilt and common scab of potato because it contains all the VFAs found in swine manure [62]. Poultry manure significantly increases yield and reduces common scab, but its consistency needs further investigation [32]. Ammonium lignosulfonate (ALS) is a nontraditional amendment that is effective in reducing potato common scab (*S. scabies*) by up to 50%, although the toxic compound is not known [26].

Some green manures can directly suppress soilborne diseases if plants contain antimicrobial substances [7,43,64,65,76,94]. Cole crops or Brassica plants produce glucosinolates that can break down into various compounds, including allyl isothiocyanates, which are toxic and effective in killing soilborne pathogens. This gas serves as a soil fumigant to inhibit or kill pathogens, and brassicas have been considered to be biofumigant crops [58,64,95]. Broccoli incorporation in soil significantly reduces *Verticillium dahliae* on cauliflower, *Sclerotinia minor* on lettuce [95,96], and *Streptomyces* spp. causing potato common scab [97]. Meanwhile, the application of Brassicaceous seed meal enhances non-pathogenic or beneficial *Streptomyces* spp. associated with the rhizosphere that produce nitric oxide (NO) and suppress *Rhizoctonia* root rot by inducing plant resistance against the disease, although the role of antimicrobial activity is not the major reason for disease suppression [58]. Sudan grass produces cyanogenic glucosides that break down into hydrogen cyanide as the active compounds for disease suppression. Incorporation of Sudan grass into soil increases microbial activities and the antagonist *Fusarium* spp. that significantly reduce *Verticillium* wilt of potato [78,98,99].

Although it is encouraging that antimicrobial substances may take effect, overall, the total amount of organic input is more important and it is positively correlated with the level of disease suppression [65,75]. In most cases, the application of green manures suppresses soilborne diseases in an indirect way. However, many studies show that under certain conditions, green manures may not significantly reduce pathogen populations in soil while disease is still suppressed [49]. This has been confirmed in apple disease systems using plant-derived products, where disease suppression was carried out by modifying the soil microbiome [100,101].

Allelopathy and antagonisms possessed by some plants can also result in the suppression of pathogen infection [102]. The chestnut (*Castanea sativa*) contains antibacterial and allelopathic compounds in leaves and fruit [102,103,104], inhibiting several bacteria and the germination. The active portion includes rutin, hesperidin, and quercetin as the most effective chemicals, as well as apigenin, morin, naringin, galangin and kaempferol [102,105]. These products can be potentially used for disease management such as for potato common scab [8].

More and more organic materials have been studied as soil amendments. Lignin promotes the efficacy of *Trichoderma* and beneficial bacteria in suppressing *Rhizoctonia solani* [60], and sawdust, bark, straws, sludge from paper mill, deciduous tree leaves, pine needles, wood chips, and phosphite suppresses common scab in potatoes [106]. Biochar is a product produced from charcoal by pyrolysis of biomass in the absence of oxygen. It is effective for disease control as it induces plant resistance against pathogens [59]. When amended into soil, biochar improves physical, chemical, and biological attributes of soils. It increases soil pH, organic matter, cation exchange capacity, and helps the efficiency of fertilizer uptake by the plant [107,108]. More importantly, biochar enriches beneficial microorganisms, such as *Bacillus* and *Lysobacter* spp. that suppress soilborne pathogens *Fusarium* and *Ilyonectria*. By modifying soil microbiomes, biochar can remove the negative effect of phytotoxicity of replant problem of ginseng (*Panax notoginseng*) [60,109].

### 3.3. Microbial Amendments—A Biological Booster of Soil Health

The soil microbiome is an ecological aggregate that contains all types of microorganisms residing in soil. Since these microbes belong to various taxa, their functions can be greatly different, varying from nutrient cycling, chemical degradation, plant growth promoting, suppressing plant growth, or causing plant infection [19,53,110]. Microbial activities are involved in the degradation or decomposition of natural or organic products, producing substances such as enzymes, hormones, and nutrient solubilizing and transporting elements, which thereby enhance plant health [38,48]. Therefore, it is crucial to enhance soil microbiome populations and their activities. In addition to plant pathogens, numerous beneficial microorganisms reside in soil and play important roles directly or indirectly contributing to plant health [81,111] (Figure 1).

There are two ways to enhance the microbial community in soil: (1) applying microbial amendments, such as adding microbial products to directly boost the population of certain taxa of microorganisms [36], and (2) applying organic matter to feed indigenous microbes and increase microbial populations in both abundance and diversity. Both methods can suppress some soilborne diseases, such as *Verticillium dahliae* and *Rhizoctonia solani* [58,64,112,113]. For using microbial amendments, specific strains or mixtures of microbes that have been well characterized can be added into soil [114]. These are referred to as “synthetic communities” [36]. The second approach is focused on the microbiome, and in contrast to synthetic communities, indigenous microbes can be boosted by adding organic amendments [32,34]. This can impact many taxa of microorganisms, so the targets may not be well defined, but such an integrated strategy may have more advantages and could be practical [42].

Bacteria producing secondary metabolites as antimicrobial substances play a key role in disease suppression. Microbial agents applied in potato production include, but are not limited to bacilli [8,115], *Trichoderma* spp., fluorescent pseudomonads [68], *Rhizobia* spp., *Lysobacter* spp., and *Streptomyces* spp. [116,117]. *Bacillus velezensis* BAC03 effectively controls common scab of potato due to the production of LCI polypeptide [8,67] and endophytic bacteria *Bacillus velezensis* strain 8–4 has a strong inhibitory effect on various pathogens, such as *Streptomyces galilaeus*, *Phoma foveat, Rhizoctonia solani, Fusarium avenaceum* and *Colletotrichum coccodes* [118]. *Bacillus subtilis* suppresses *Streptomyces scabies* by producing AMEP412, a protein elicitor with antimicrobial activity [119]. The degradation fragments of gamma–glutamyl transpeptidase from *Bacillus subtilis* BU108 have antimicrobial activity against *Streptomyces scabies* [119], and secondary metabolites such as surfactin, iturin A, and fengycin are commonly reported as antimicrobial substances [120]. *Pseudomonas synxantha* LBUM223 applied in the field suppresses common scab of potato [68]. The bacterium produces phenazines that have been used as an indicator of disease suppression [121]. Non-pathogenic *Streptomyces* strains can suppress pathogenic species *S. scabies* [65] or *S. galilaeus* [116].

Various types of fungi have been studied for potential biological control [122,123,124]. *Trichoderma virens* have been used and partially effective for the control of black scurf and common scab, which also shifts substance utilization when applied with composts [30,47]. The hypovirulent strain of *R. solani*, Rhs1A1 added in soil significantly affected microbial community structure, microbial activity and bacterial abundance [47], possibly due to increased plant growth and root biomass associated with the plant growth-promoting Rhs1A1 [125]. Rhs1A1 amended in soil also increases the populations of *Trichoderma* spp. because a portion of the Rhs1A1 population may be parasitized by *Trichoderma* species [115]. Additionally, mycorrhiza help water and mineral absorption, and many mycorrhizal products have commercially been applied in potato production [126,127,128].

In addition to direct inhibition or antagonism of plant pathogens, many biological control agents have multiple roles, including inducing plant resistance and increasing yield [129,130,131]. *Pseudozyma aphidis* possesses a direct inhibitory effect, but its major role is to induce plant resistance against *Botrytis cinerea* by inducing jasmonic acid and salicylic acid/NPR1 [69]. Some products have strong effects in enhancing plant growth and are primarily used as a biofertilizer [36]. The plant growth-promoting rhizobacterium *Bacillus velezensis* strain BAC03, in addition to producing the antimicrobial substance LCI, produces plant hormones such as indole–3–acetic acid (IAA), NH_3_, acetoin and 2,3–butanediol, and has 1–aminocyclopropane–1–carboxylate deaminase activity in promoting plant growth [132]. Therefore, disease suppression and plant growth promotion are two main roles in most biocontrol agents.

Although soil amendments are subjectively divided into three groups, there are no distinct lines between them because there is some overlap. For example, clover can be used as a rotation or cover crop, and its endophytes contain some bacteria that help plants to play an allelopathic function [133]. In the 1950s, Menzies [134] observed that adding alfalfa meal consistently strengthened the suppressiveness of soil to common scab through promoting disease-suppressive microbes. This may be an early example of organic matter input that drives the microbial activities of soil in disease control. Microorganisms affect allelopathic activities by degrading allelochemicals from plants [105]. Specific microorganisms may be directly added with well characterized functions, but the majority of functional microbes that take effect come from the soil as indigenous microbial communities that may be augmented by added organic amendments.

## 4. Measuring Soil Health

When soil amendments are applied, it is essential to qualify and quantify the effect of the soil treatment, and there are different indicators that have been used [39]. These include soil physical and biochemical properties, microbial communities, and disease suppression indicated by reduced populations of pathogens and incidence of disease. For soil property analysis, standard procedures have been well established and applied. Soil pH, N content and cations and oligoelements can be indicators [39]. Since there are many parameters that contribute to soil health at different levels, metadata can be analyzed using principal component analysis (PCA) or similar statistical methods.

For microbial analysis, soil health can be measured by microbial biomass. For example, the enzyme activity of microbes is measured using fluorescein diacetate (FDA) hydrolysis assays [34]. For microbial enumeration or isolation, culture-based methods have been used with various semi-selective media [81]. Specific groups of microbes can be observed and isolated by eliminating and suppressing other organisms. The culture-based method has limitations. Less than 5% total microbial organisms can be identified or analyzed because most of them in soil are not culturable. In addition, some other methods such as fatty acid methyl ester profile (FAME) and substrate utilization analysis with BIOLOG assays are alternatives to profiling microbial communities, although they are less used in more recent studies [34]. The obtained or isolated microbes can be further characterized by biological and genetic analyses.

To overcome the low level of taxonomic capacity, high throughput sequencing based methods or metagenomic approaches have greatly enhanced our capability in measuring the totality of microbiomes, and almost all taxa can be detected and analyzed [135,136,137,138,139,140]. Sequence data are processed using bioinformatic analyses, which provides microbial diversity, abundance, and functional relationships. By combining sequence– and culture-based methods, soil microbial communities can be well characterized [81,140].

By employing multiple methods in studies, the effect of soil amendments can be analyzed with fine resolution. For example, functional genes related to antimicrobial activities can be assayed using quantitative real-time polymerase chain reaction [100,101], antimicrobial substances and secondary metabolites can be detected using high performance liquid chromatography coupled with mass spectrum analysis [141,142]. In addition to metagenomics, new omics tools have made it possible for more rapid screening of beneficial microbes having multiple functional attributes that may contribute to pathogen suppression, such as transcriptomics to measure mRNA transcript level, proteomics to quantify protein levels, metabolomics to measure abundance of cellular metabolites, and interactomics to determine molecular interactions [36,143,144].

## 5. Challenges of Applying Soil Amendment

Although it is advantageous to use soil amendments, there are challenges in disease control and the effects are not consistent [31,32,47]. This can be improved through further studies on mechanisms of how amendments impact soil and how they vary as environmental conditions change. de Medeiros et al. found from 20 published studies that in using biochar, 70% are positive in controlling plant diseases, 10% vary depending on soil conditions, and 20% are not effective against *Pythium ultimum* and *Rhizoctonia solani* [59]. Although there is not always significant yield benefit, biochar may contribute in some other ways, such as in the absorption of toxins or heavy metals [109], in the interaction with microbes, and in improving soil carbon sequestration [60,109]. Increasing yield and reducing disease are often related, but can be affected by different mechanisms. Since most organic amendments are rich in carbon or nitrogen, it is not surprising that their application can increase plant yield. However, they may not be highly effective in disease suppression. For example, incorporating millet into soil had little effect on the reduction in Verticillium wilt, but significantly increased potato yield up to 50% [49]. Similarly, although seed meals from *Brassica* spp. may have antagonistic effects on disease suppression, they may not be highly effective to inhibit all pathogens, and sometimes can increase the population of certain organisms, such as *Rhizoctonia* spp. [28].

In applying crop rotation, a longer crop rotation leads to better disease control; however, this is not always practical for economic purposes. In addition, it can be challenging to select rotation crops because there are limited sources of crops that are not infected (non-hosts) and some plants used for rotation with potato may be susceptible to other non-target pathogens. For example, barley, cotton and sugar beets do not reduce, but rather increase common scab with each successive crop of potatoes [66]. Some plants (non-hosts) not susceptible to certain pathogens can harbor or increase the population of these pathogens during the growing season, such as cowpea, riverhemp, and maize, which can increase *Streptomyces scabies* [55]. The application of green manure can suppress *Rhizoctonia* spp. and recovery of *Pratylenchus* spp., but increases *Pythium* population [58]. Ultimately, there is no one size fits all formula in the use of amendments. Therefore, knowing the mechanisms of a specific product, will help us to consistently add function in enhancing soil and plant health.

Many fungi are useful in disease suppression; however, they may also pose risks because they can affect non-target fungi or other beneficial microorganisms [145]. Another example is *Streptomyces* spp. in soil, which is a large genus containing various types of species in terms of functions. Typically we can find many species causing common scab of potato [31,114], but there are also many species that have antagonistic and antimicrobial activities, which is beneficial to potatoes [81,116]. Because these bacteria have similar genetic backgrounds and biology, providing bulk organic matter may not specifically enhance only beneficial species, but also pathogens in this taxon as well. The pros and cons of this system should be carefully studied and considered to ensure the proper outcomes are achieved.

The application of soil amendments can be evaluated for soil health by measuring various biochemical, biological, and physical variables [19]. Conventionally, soil quality and soil health have been measured by analyzing soil fertility and physiological properties, abiotic parameters including pH values, N, and C contents [52]. Analysis of the microbiome has been used as an indicator and measurement of soil health [43], which has shown to be a powerful tool using new available technologies [42]. Microbial population can be estimated based on culturing methods, biomass microbial diversity and abundance, and microbial activity [29,39], as biological indicators such as microbial biomass, enzymatic activities, metabolomic activities, and organic matter. These have been a standard to determine the integrity of plant health by analyzing the soil microbiome in diversity, abundance, and other parameters. Higher abundance and diversity levels are always associated with healthy soil because a complicated network of microorganisms provide different functions. The microbial communities can then be further divided, such as into beneficial organisms and pathogens [146].

Omics approaches have greatly enhanced microbial studies. Data libraries established from previous studies provide a foundation for taxonomy and functions of microbiome, such as whole genome sequences of both microorganisms and plants, microRNAs of plants in response to biotic and abiotic stresses, and networks [144,147]. As metadata expands quickly, machine learning can be utilized for analyzing complicated networks.

## 6. Conclusions 

Soil health is determined by whether potential physiological conditions are met and whether potential plant pathogens are eliminated or suppressed. A simple implementation of soil amendments has multiple benefits in improving soil health and potato health. It can perform a “two birds with one stone” function. Added organic matter either directly impacts pathogens and plants or can be used as energy for soil microorganisms that in turn suppress plant diseases and provide nutrients to plants. The goal of disease management is not to kill any detrimental organisms but to adjust the balance of microbial communities so that pathogens are suppressed by other microbial forces. To enhance the microbial activity, some beneficial microbes with strong antagonistic characteristics can be applied either individually or as mixed species of microorganisms added to soil. As our knowledge and technologies advance, we will have a much better understanding and ability to utilize amendment-based strategies. The future direction of research should be on understanding the mechanisms of amendment and their corresponding impact on specific environments, plants, and pathogen conditions.

## Figures and Tables

**Table 1:** Examples of soil amendments used for improving soil health based on individual studies.

Type of Input	Target Disease	Potential Mechanism to Reduce Diseases	Source
**Organic Amendment**			
*Brassica napus* seed meal	*Rhizoctonia solani*	Change in soil microbial communities that induce plant resistance	[58]
Biochar	Various pathogens	Induced plant resistance, improve soil properties and microbial growth, toxin immobilization and transformation	[59,60]
Blood meal	*Verticillium dahliae*	Ammonia, nitrous acid	[49]
Swine manure	*Verticillium dahliae Streptomyces* spp.	Volatile fatty acids—ammonium lignosulfonate	[61]
Ammonium lignosulfonate	*Verticillium dahliae*	Antifungal effect	[26]
Fish emulsion	*Verticillium dahliae* *Streptomyces* spp.	Organic acids, toxic compounds	[62]
Compost	*Rhizoctonia solani*	Increased utilization of complex substrates and increased levels of Gram-positive bacteria and fungi	[30,47]
**Rotation**			
Barley/ryegrass	*Rhizoctonia solani*	Enhanced soil microbial activities in disease suppression	[7,53]
Red clover or Barley undersown with red clover	*Rhizoctonia solani* *Phytophthora erythroseptica*	Pathogen suppression	[54,63]
Mungbean and Sunn hemp	*Streptomyces scabies* Nematodes	Pathogen suppression and enhancing beneficial microorganisms	[38,55]
Broad bean	Non-specific	Enhancing soil microbial communities, diversity and crop yield	[56]
**Green Manure/Cover Crop**			
Mustard	*Verticillium dahliae* and other soilborne diseases	Antimicrobial activities	[64]
Brassica	*Rhizoctonia solani* and other soilborne diseases	Antimicrobial activities	[7]
Sunn hemp	Common scab (*Streptomyces* spp.)	Pathogen suppression	[55]
Buckwheat	Verticillium wilt	Modifying antagonistic streptomycetes	[65]
Soybean	Common scab	Pathogen suppression	[65,66]
**Microbial Amendment**			
*Bacillus Velezensis*	Common scab (*Streptomyces* spp.)	Plant resistance inducing, LCI protein and volatile Organic compounds for antimicrobial activity, hormones promoting plant growth	[8,67]
*Pseudomonas* *fluorescens*	Common scab (*Streptomyces scabies*)	Produces Phenazine–1–Carboxylic (PCA) production as antimicrobial substance	[68]
*Pseudozyma aphidis*	*Botrytis cinerea*	Antimicrobial activity, induced plant resistance by inducing jasmonic acid and salicylic acid/NPR1	[69]

Note: different soil types/climatic and soil conditions could produce varied results. Therefore, we only demonstrate some studies that show positive results in improving soil health and suppressing diseases.
